# Microbial Reprogramming in Obsessive–Compulsive Disorders: A Review of Gut–Brain Communication and Emerging Evidence

**DOI:** 10.3390/ijms241511978

**Published:** 2023-07-26

**Authors:** Ghizlane Bendriss, Ross MacDonald, Clare McVeigh

**Affiliations:** Weill Cornell Medicine Qatar, Doha P.O. Box 24144, Qatar

**Keywords:** OCD, obsessive–compulsive disorder, microbiota, gut, gut–brain axis, probiotics, fecal transplants, microbial reprogramming

## Abstract

Obsessive–compulsive disorder (OCD) is a debilitating mental health disorder characterized by intrusive thoughts (obsessions) and repetitive behaviors (compulsions). Dysbiosis, an imbalance in the gut microbial composition, has been associated with various health conditions, including mental health disorders, autism, and inflammatory diseases. While the exact mechanisms underlying OCD remain unclear, this review presents a growing body of evidence suggesting a potential link between dysbiosis and the multifaceted etiology of OCD, interacting with genetic, neurobiological, immunological, and environmental factors. This review highlights the emerging evidence implicating the gut microbiota in the pathophysiology of OCD and its potential as a target for novel therapeutic approaches. We propose a model that positions dysbiosis as the central unifying element in the neurochemical, immunological, genetic, and environmental factors leading to OCD. The potential and challenges of microbial reprogramming strategies, such as probiotics and fecal transplants in OCD therapeutics, are discussed. This review raises awareness of the importance of adopting a holistic approach that considers the interplay between the gut and the brain to develop interventions that account for the multifaceted nature of OCD and contribute to the advancement of more personalized approaches.

## 1. Introduction

Obsessive–compulsive Disorder (OCD) is a chronic mental health disorder characterized by the presence of intrusive and persistent thoughts that cause distress called obsessions; these are followed by compulsions, which are repetitive behaviors or mental acts that individuals feel driven to perform to calm their obsessions [1]. OCD affects approximately 2–3% of the global population and greatly interferes with quality of life, disturbing the daily functioning of an individual, from eating to bathing to walking or even breathing. For example, an individual with OCD can spend thirty minutes closing a door and verifying it is closed, with the hope that the anxiety might be calmed after a certain number of repetitions. The decision making of individuals with OCD is greatly affected, as every decision may be felt as a threat, leading to maximum indecisiveness [2,3,4]. It is a very debilitating disorder that often hides behind another disease or disorder. Indeed, although OCD is now recognized as an independent disorder category, it often occurs with other disorders such as autism, attention deficit hyperactivity disorder (ADHD), depression, general anxiety disorder, eating disorder, hoarding disorder, Tourette syndrome, panic disorder, or schizophrenia [5]. This category includes other disorders such as hoarding disorder, hair-pulling disorder, and skin-picking disorder [6].

The exact mechanisms underlying OCD are not yet fully understood. Research has highlighted several associations, leading to the conclusion that a combination of genetic, neurobiological, immunological, and environmental factors may contribute to its development. Indeed, studies have identified the heritability of OCD through multiple genes such as the serotonin transporter gene (SLC6A4) and the gene encoding the dopamine D2 receptor (DRD2) [7,8]. Also, OCD has been associated with neurobiological changes such as the dysregulation of the cortico-striato-thalamo-cortical (CSTC) circuit. Brain regions involved include the orbitofrontal cortex, the anterior cingulate cortex, and the basal ganglia, as well as dysregulation in neurotransmitters like serotonin, dopamine, and glutamate [6,9,10,11]. In addition, environmental factors, such as childhood trauma, including physical and/or sexual abuse, have been associated with an increased risk of developing OCD. Finally, stressful life events, such as significant life changes or trauma, have been found to precede the onset or exacerbation of OCD symptoms [6,12].

To date, cognitive behavioral therapy (CBT) and pharmacotherapy are the primary treatments for OCD [13,14]. CBT typically involves exposure and response prevention, where individuals are gradually encouraged to face their obsessions while refraining from engaging in their compulsive behaviors. This helps to reduce the anxiety associated with the obsessions and weaken the link between the obsession and compulsion. CBT has been shown to be effective in reducing OCD symptoms and improving overall functioning [14]. On the other hand, selective serotonin reuptake inhibitors (SSRIs), such as fluoxetine, sertraline, and fluvoxamine, are the first-line medications for OCD treatment [13,15]. These medications increase serotonin levels in the brain and help alleviate symptoms. Additionally, combining SSRIs with antipsychotics or glutamate modulators is sometimes used for individuals who do not respond adequately to SSRIs alone [16]. Despite the availability of treatment options for OCD, there are significant limitations that warrant the exploration of novel therapeutic approaches. While these interventions can be effective for some individuals, many patients experience only partial responses to treatment, lingering symptoms, and high rates of relapse [16,17]. CBT is efficient, but each treatment plan is specific to an obsession and does not avoid the appearance of another obsession and compulsion later, which would require another course of CBT. Additionally, there are side effects associated with the use of medication, such as gastrointestinal disturbances and sexual dysfunction, which can further impact treatment adherence and quality of life [18].

The limitations of current treatment options emphasize the need for innovative and therapeutic approaches that target the etiology of OCD. To date, several factors have been proposed to contribute to the development of OCD, and it is difficult to point to one single cause. Nevertheless, there is one emerging avenue of investigation that presents itself as promising and key for the understanding and treatment of OCD: the gut microbiota. The gut microbiota comprises trillions of microorganisms residing in the gastrointestinal tract, from bacteria to fungi to viruses, archaea, and protozoa. These microbes outnumber human cells by a factor of 10, and the genes they express form the microbiome [19]. These are usually classified into three categories according to their interaction with their human hosts: beneficial, pathogens, and commensal microbes. Because they control each other’s growth, eubiosis (the undefined but balanced composition of the gut microbiota) is essential to prevent the overgrowth of pathogens or a lack of growth of certain beneficial microbes from lacking. In contrast, dysbiosis refers to an imbalance in the composition or function of the gut microbiome. It can occur when there are changes in the relative abundance of certain microbial species or alterations in the overall diversity, resulting in the alteration of the metabolites produced by the microbiota. Dysbiosis has been associated with various health conditions, including metabolic disorders, mental health disorders, autoimmune diseases, and inflammatory bowel diseases [20,21,22,23,24,25,26,27,28,29,30,31,32,33]. Interestingly, dysbiosis has been associated with all disorders where OCD has been found as a comorbidity such as autism, Tourette Syndrome, anxiety disorders, panic disorder, eating disorders, depression, and hoarding disorder but also gastrointestinal diseases such as ulcerative colitis and Crohn’s disease [34,35,36].

While we acknowledge that no study has pointed to the prevalence of the co-occurrence of dysbiosis and OCD in anxiety disorders, the latest advances in the understanding of bidirectional communication between the gut and the brain strongly implicate the gut microbiome as a key component for future investigations. In this review, we examine the growing evidence that supports the possible causal role of dysbiosis in these anxiety disorders. We also discuss the emerging clinical studies that aim to modulate the gut microbial composition to increase its diversity and inhibit the growth of pathogens.

We will first review the molecular mechanisms involved in the microbiota–gut–brain axis (MGBA). Then, we will gather the latest evidence that supports our rationale and the latest evidence that shows dysbiosis in OCD and how dysbiosis fits into a model explaining the neurochemical, genetic, immunological, and environmental basis of OCD. Finally, we will review recent clinical interventions that support the promising potential of two microbial reprogramming strategies: dietary interventions using prebiotics and probiotics and fecal microbiota transplantation (FMT). We will discuss the challenges of studying such clinical interventions in OCD and identify important considerations for future clinical studies.

## 2. Mechanisms of the Microbiota–Gut–Brain Axis (MGBA)

**Figure 1:**
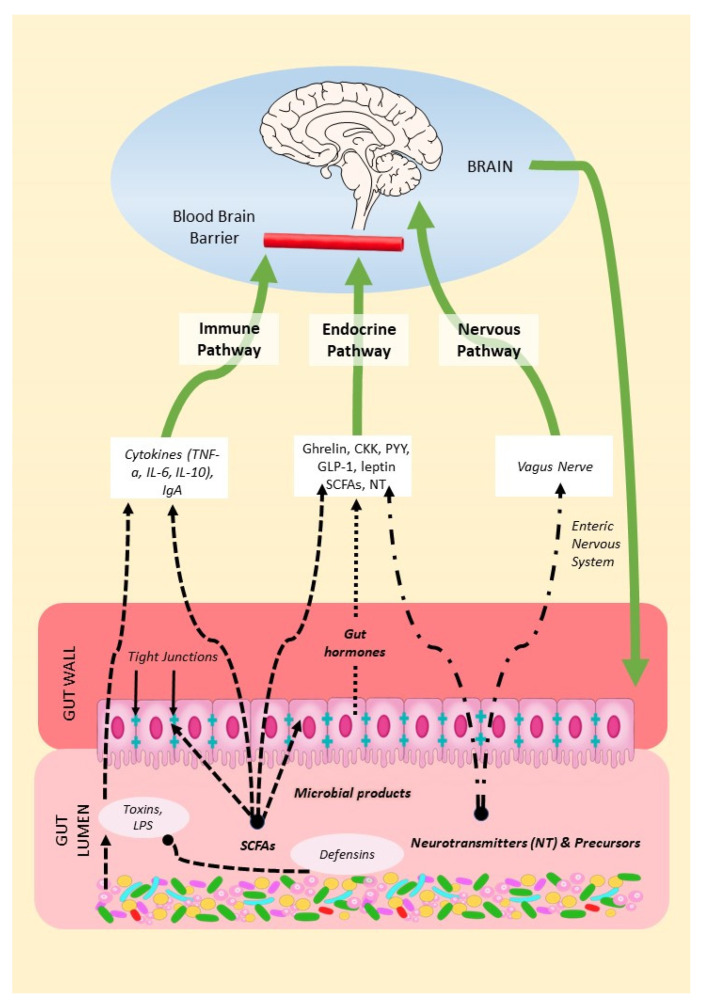
Mechanisms of the MGBA. Gut microbes in the gut lumen produce SCFAs, neurotransmitters, precursors, and defensins but also toxins. Microbial products can thus modulate gut permeability and immune function, endocrine secretions, and the vagal function of the brain.

The MGBA refers to the bidirectional communication between the gut microbiota, the gastrointestinal tract, and the central nervous system (CNS). From brain to gut, endocrine systems such as the hypothalamic–pituitary–adrenal and hypothalamic–pituitary–gonadal axes regulate the gut microbiota [37,38,39]. From gut to brain, the gut microbiome, consisting of microbes, their genomes, and their products, can influence brain function through a variety of mechanisms, summarized in Figure 1. We will describe these below as (1) the endocrine pathway; (2) the nervous pathway; and (3) the immune pathway.

### 2.1. The Endocrine Pathway

The endocrine pathway involves the release of microbiota-derived products into the systemic circulation, issued by the metabolic activity of microbes, including short-chain fatty acids (SCFAs), neurotransmitters, hormones, and inflammatory factors that directly or indirectly regulate the function of the CNS.

The production of SCFAs by gut bacteria after the anaerobic fermentation of indigestible polysaccharides, such as dietary fibers and resistant starch, plays a crucial role in modulating the metabolic activity of the gut, and they are pivotal in microbiota–gut–brain crosstalk [40,41,42]. A variety of SCFAs are produced depending on the nature of the dietary fibers being digested: the most abundant are butyrate, acetate, and propionate [43,44].

Following their production, SCFAs can cross the enterocyte layer and be absorbed by colonocytes. This happens mainly via H^+^-dependent or Na^+^-dependent monocarboxylate transporters [44]. They can regulate gut barrier integrity and mucosal immunity through various molecular mechanisms involving G protein-coupled receptors such as free fatty acid receptors 2 and 3 or hydrocarboxylic acid receptors [45]. Butyrate has been shown to promote the upregulation of proteins constituting tight junctions, such as zonula occludens-1, claudin-5, and occulin, and the inhibition of zonulin to reduce intestinal permeability and maintain gut barrier integrity [46,47,48,49,50,51,52]. A decrease in the abundance of butyrate can lead to leaky gut syndrome, thereby influencing the immune response, as well as the integrity of both the gut and the blood–brain barrier (BBB) [53,54,55,56,57]. Indeed, the expression of claudin and occludin has also been shown to be reduced in the BBB of germ-free mice, leading to the increased permeability of the BBB from intrauterine life to adulthood [58]. The brain’s uptake of SCFAs has previously been shown in rats [59], and studies have shown detectable levels of acetate, propionate, and butyrate in their cerebrospinal fluid [60]. In another study, the brains of mice supplemented with live *Clostridium butyricum* had significantly higher concentrations of butyrate than did peripheral blood [61,62]. The recolonization of these adult mice with complex microbiota or monocolonization with SCFA-producing bacterial strains recovered the integrity of the BBB [56,58]. Similarly, the treatment of an in vitro model of cerebrovascular endothelial cells with propionate attenuated the permeabilizing effects of exposure to lipopolysaccharide (LPS) [63].

Sometimes, protein fermentation in the distal portion of the intestine can lead to the production of potentially toxic metabolites, such as ammonia, phenols, and sulfides, as well as unique branched-chain fatty acids [64,65,66,67]. By controlling BBB integrity, SCFAs play a pivotal role in the passage of these and other molecules and nutrients from the circulation to the brain, playing a central role in brain development and the preservation of CNS homeostasis [57,68,69,70].

### 2.2. The Nervous Pathway

Various gut bacteria have been shown to also produce neurotransmitter precursors and hormones, such as dopamine, acetylcholine, γ-aminobutyric acid, noradrenaline, serotonin, and corticotrophin-releasing hormone [71]. In addition to producing peripheral serotonin, gut microbes can affect the transmission of central serotonin by modulating the production of tryptophan in plasma. This has been demonstrated for *Bifidobacterium infantis* [72]. Enterochromaffin cells can bind several microbial products and secrete serotonin into the lamina propria, increasing colonic and blood concentrations of 5-HT [73,74]. SCFAs regulate the expression levels of tryptophan 5-hydroxylase 1—the enzyme involved in the synthesis of serotonin—and tyrosine hydroxylase, which is involved in a rate-limiting step in the biosynthesis of dopamine, noradrenaline, and adrenaline. SCFAs thereby exert an effect on brain neurochemistry [73,74,75,76]. The neural pathway involves bidirectional communication between the gut and the brain via the autonomic nervous system or the vagus nerve [77,78,79,80]. The vagus nerve serves as a major conduit for transmitting signals between the gut and the CNS. When gut bacteria modulate the production of neurotransmitters such as γ-aminobutyric, serotonin, dopamine, norepinephrine, glutamate, and acetylcholine, these can bind to the primary afferents of the enteric nervous system and the vagus nerve to influence brain function and behavior. Retrograde transport also plays an important role in gut-to-brain communication. It is now recognized that the onset of Parkinson’s disease probably starts in the gut, with α-synuclein aggregation upon LPS binding and the retrograde transport of aggregates through the vagus nerve to the brain. There is accumulating evidence that SCFAs may also modulate key neuropathological processes underlying Alzheimer’s disease by interfering with the assembly of amyloid-β peptides into neurotoxic oligomers [81,82,83]. In addition, the metabolites of gut microbes, by controlling the secretion of gut hormones such as glucagon-like peptide 1 and peptide YY, can influence food intake, which will, in turn, influence bacterial fermentation, thereby reinforcing the close relationship between diet and gut microbiomes [84].

### 2.3. The Immune Pathway

#### 2.3.1. From Gut to Host Immune System

Changes in the gut microbiota composition can affect the production and availability of SCFAs, thus impacting the metabolic activity of the gut [85,86,87,88,89]. Dysbiosis, by leading to reduced SCFA production, impaired gut barrier function, and increased intestinal permeability, is a starting point for systemic inflammation and potential neuroinflammation [90,91]. The byproducts of microbiota metabolism can activate immune cells in the gut, leading to the production of pro-inflammatory or anti-inflammatory cytokines [92,93,94,95,96]. These immune signals can then communicate with the brain and affect neural function, as neuroinflammation is an important process shaping brain function.

A good example of this is bacterial lipopolysaccharides (LPS), also known as endotoxins, which are components of the outer membrane of Gram-negative bacteria. They are typically produced as part of the bacterial growth and replication process [97]. These endotoxins trigger a pro-inflammatory cascade in the mucosa, mediated by toll-like receptor 4 and cytokines such as tumor necrosis factor α (TNF-α) and interleukin 6 (IL-6) [97]. The LPS-induced pro-inflammatory cascades have been shown to be inhibited by the butyrate inhibition of histone deacetylase (HDAC) [98]. This intracellular signaling has been found not only in the gut and associated immune tissue but also in the peripheral nervous system and CNS [51,99,100,101,102]. Perturbations of the gut microbiota caused by antibiotics in experimental animal models systemically produced altered immune responses with pro-inflammatory profiles [103]. In early life, if the microbiota is depleted using antibiotics, this results in an inflammatory response in the CNS with pro-inflammatory cytokine secretion and altered microglial morphology, which could be reversed by butyrate treatment [104,105,106,107,108,109,110]. Indeed, butyrate has been shown to control the maturation of mucosa-associated lymphoid tissue and the differentiation of lymphocytes, characterized by the presence of macrophages and B and T cells. Similarly, acetate treatments of microglia primary culture in vitro have been shown to reduce inflammatory signaling by downregulating the expression of IL-1β, IL-6, and TNF-α and the phosphorylation of p38 MAPK, JNK, and NF-κB [111]. The precise signaling involved in the effects of SCFAs on microglia remains unclear, and histone acetylation or epigenetically regulated gene expression is considered the main mechanism [112].

#### 2.3.2. From Host Immune System to Gut Microbiota

Several studies investigating the relationship between immunoglobulin A (IgA) and the gut microbiome have concluded that the adequate production of IgA is essential for the colonization of certain “good” bacteria such as Bifidobacterium and Bacteroides [113,114,115,116,117,118,119,120,121]. On the other hand, microbial acetate produced by gut microbes is also able to regulate IgA reactivity to commensal bacteria, thus highlighting a bidirectional relationship between gut microbes and the immune system [70,122,123,124,125].

IL-22 and IL-17 have been shown to stimulate gut intraepithelial cells into producing antimicrobial peptides such as α-defensins and β-defensin 1, which can quickly inactivate microorganisms entering the host through a leaky gut [126,127]. Furthermore, mice transgenic for defensins have exhibited an altered microbiota composition [128,129,130,131]. Thus, antimicrobial peptides affect the microbial composition.

## 3. Dysbiosis and the Neurobiology Basis of OCD

### 3.1. Dysbiosis in OCD

While there is a wealth of research associating the gut microbiome with other neuropsychiatric disorders that may involve OCD-like behaviors, such as autism and ADHD, studies exploring the gut microbiome in OCD specifically remain scarce [132,133,134]. Nevertheless, the potential role of the gut–brain axis in the pathophysiology of OCD has been highlighted by several studies, suggesting that alterations in gut microbiota composition may impact brain function and behavior, including obsessive–compulsive symptoms. Our search through PubMed, Scopus, and Embase yielded many reviews but only a few emerging clinical studies. Table 1 lists clinical studies that specifically looked at the gut microbiota in OCD, most of them published in the past two. Although clinical interventions targeting dysbiosis and focusing specifically on OCD are scarce, a few studies are worth mentioning. A recent scientific report by Domenech et al. (2022) reported dysbiosis in the gut and oropharyngeal microbiomes of OCD patients [135]. They noted an increase in bacteria from the *Rikenellaceae* family, associated with gut inflammation, and a decrease in bacteria from the *Coprococcus* genus. Lower bacterial diversity in the gut of OCD patients has been observed, consistent with the lower gut α-diversity in PANS/PANDAS patients reported by Quagliariello et al. [136] and in OCD by Turna et al. [137]. The latter observed a decrease in species richness/evenness and a lower relative abundance of three butyrate-producing genera (Oscillospira, Odoribacter, and Anaerostipes) in OCD patients [138]. Furthermore, lower α-diversity has also been reported in subjects with ADHD [139,140,141] and in studies of ASD individuals [28,29,142,143,144,145]. Despite the small sample sizes and the variability in methodologies used, the studies presented in Table 1 highlight the emerging field of study in the treatment of OCD [135,136,137,138,146,147,148,149,150].

These findings not only demonstrate the importance of further exploring the gut–brain axis in OCD but also suggest a possible causal link between changes in the gut microbiota and the development of obsessive–compulsive-like behaviors. Overall, while there is emerging evidence suggesting a correlation between gut microbiota dysbiosis and OCD, there is a genuine need for further research specifically investigating the relationship between OCD and the microbiota before establishing a causal relationship and determining the clinical implications.

**Figure 2:**
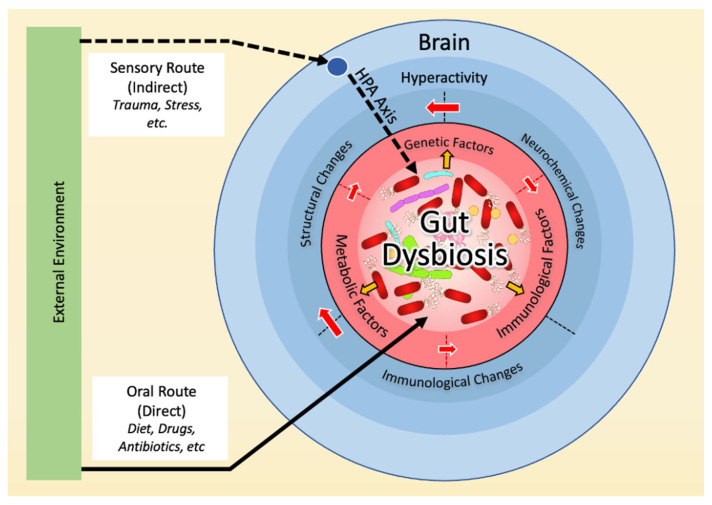
Hypothetical model of the microbiological basis of OCD. A direct (oral) and an indirect (sensory) pathway can affect the gut microbial composition, which, in turn, can modulate (yellow arrows) immunological, genetic, and metabolic factors in the host. Those factors interact with each other (red arrows), resulting in structural, neurochemical, and immunological changes in the brain, leading to hyperactivity.

The below hypothetical model places gut dysbiosis at the center of all factors previously associated with OCD, interacting with the neurochemical, immune, genetic, and environmental bases of OCD (Figure 2). Below, we will detail evidence in support of this model.

### 3.2. Dysbiosis and Hyperactivity in the Cortico-Striato-Thalamo-Cortical Circuit (CSTC)

The CSTC projects from the orbito-frontal-cortical region (OFC) to the striatum and then onward to thalamic sites before looping back to the cortex. It is responsible for reward- and motivational-related processes, executive function, motor and response inhibition, and habit-based behavior [151]. Two pathways within this circuit, direct and indirect, should have opposing net effects on the thalamus, and this balance is critical for the initiation and suppression of behavior [151]. Any imbalance is thought to contribute to OCD pathology. Indeed, overactivity in the direct pathway results in hyperactivity in the feedback loop, creating overall hyperactivity within the circuit. Several studies have noted an increased activation of the OFC and the striatum and caudate regions [6,152,153,154,155,156,157,158,159,160]. This hyperactivity is believed to involve the neurotransmitters serotonin, glutamate, and dopamine [76,153,161].

#### 3.2.1. Serotonin

Adams et al. (2005) pointed to the increased binding of 5-HT2AR, the receptor to serotonin, in the caudate nuclei of unmedicated OCD patients, possibly due to the compensatory effects of low levels of serotonin within the CSTC circuit [154]. Simpson et al. (2020) showed that an earlier onset of OCD was associated with increased 5-HT2AR availability in the circuit [155]. This is supported by successful treatments using serotonin receptor inhibitors (SRI). Nevertheless, mechanisms through which SRIs ameliorate symptoms are still not well understood, and only 40–60% of patients improve following SRI intervention [156,157].

The gut microbiota plays a crucial role in the production of serotonin. Specific gut bacteria, such as the Lactobacillus and Bifidobacterium species, have been found to produce serotonin in the gut [158]. Changes in the composition of the gut microbiota can disrupt this serotonin production process, leading to imbalances in serotonin levels. Certainly, several gut bacteria (such as Clostridium, Burkholderia, Streptomyces, Pseudomonas, and Bacillus) play a role in the metabolism of tryptophan [153,158,159]. Although in normal conditions peripheral serotonin cannot freely cross the BBB, its precursor, tryptophan, can cross the BBB through specialized transport mechanisms to then be converted into serotonin by local neurons. Furthermore, dysbiosis can both influence the availability of tryptophan and disrupt the BBB, allowing other molecules inside the brain. Certain gut bacteria can metabolize tryptophan, affecting its availability for serotonin production [153,159,160,161,162,163]. Changes in gut microbial composition can alter tryptophan metabolism, potentially impacting serotonin levels in the brain. As a result, dysbiosis-induced alterations in the gut microbiota could result in reduced serotonin synthesis in the brain, potentially contributing to mood disorders and behavioral changes.

Gut dysbiosis can also influence the serotoninergic system in the brain through other mechanisms. For instance, the gut microbiota can affect serotonin signaling by influencing the expression and activity of serotonin receptors in the brain. Dysbiosis-related changes in the gut microbial composition have been associated with alterations in serotonin receptor expression and function. A study by Hsiao et al. (2013) [164] explored the impact of gut dysbiosis on serotonin signaling. They investigated mice with gut microbiota imbalances and observed abnormal serotonin receptor expression patterns in specific brain regions. These changes were associated with behavioral abnormalities, including altered social interactions and increased anxiety-like behavior. This suggests that dysbiosis-induced disruptions in the gut microbiota can influence serotonin receptor function, potentially contributing to mood disorders and behavioral dysregulation.

#### 3.2.2. Glutamate

The glutamate system is the major excitatory neurotransmitter system in the brain. Studies have shown that untreated OCD patients have elevated glutamate concentrations in the caudate region as compared with healthy individuals; these normalized after 12 weeks of SRI treatment, suggesting that the availability of serotonin at the frontal region of the circuit might modulate the concentration of glutamate in the caudate part [165]. Because there are important glutamatergic projections between the frontal cortical part and the striatum, it was proposed that the SRI treatment allowed for an increase in serotonin levels that, in turn, inhibited glutamate levels in the caudate. In contrast, with low levels of available serotonin, the inhibitory effects within the circuit are reduced, which would allow for elevated glutamate activity in the circuit [165,166]. Increased glutamate concentrations have also been observed in the cerebrospinal fluid (CSF) of untreated OCD patients [167]. Unsurprisingly, the modulation of glutamate via n-acetyl cysteine showed improvements in double-blind placebo-controlled studies for the obsessive–compulsive-related disorders trichotillomania and skin-picking disorder [168,169].

The gut microbiota has been shown to influence the glutamate system through two main mechanisms. Firstly, certain gut bacteria, such as the Lactobacillus and Bifidobacterium species, are capable of producing and metabolizing glutamate, thereby influencing its levels in the body. Dysbiosis-induced changes in the composition of the gut microbiota can lead to alterations in glutamate production and metabolism, potentially impacting glutamate signaling in the brain. The significance of gut bacteria in glutamate metabolism has been demonstrated [170,171]. Specific bacterial enzymes have been identified that are involved in glutamate production, as germ-free mice lacking these bacteria have lower levels of brain glutamate compared with control mice, indicating the influence of specific gut bacteria on brain glutamate levels.

Secondly, dysbiosis can affect the expression and function of glutamate receptors and transporters in the brain. Changes in the gut microbiota can lead to modifications in the expression of glutamate receptors, such as N-methyl-D-aspartate (NMDA) receptors, and glutamate transporters, such as EAAT3. In their investigation of the impact of gut dysbiosis on glutamate-related pathways, Sharon et al. (2019) found that mice with disrupted gut microbiota exhibited altered NMDA receptor and EAAT3 transporter expressions in the brain, along with neurobehavioral abnormalities [110]. These findings suggest that dysbiosis-induced changes in the gut microbiota can influence the function of glutamate receptors and transporters, potentially influencing glutamate neurotransmission in the brain.

#### 3.2.3. Dopamine

Imaging studies have revealed increased dopamine levels in the basal ganglia of OCD patients and enhanced binding to the dopamine transporter [172]. One study found increased density in the dopamine transporter of the left caudate and the left putamen of untreated OCD patients [173]. The antipsychotic drugs that are sometimes offered to OCD patients who resist SRI treatments block subcortical dopamine receptor activity and are proposed to target the habit system and compulsive behaviors.

The gut microbiota can also influence the dopamine system. Some gut bacteria, including certain strains of Enterococcus and Lactobacillus, are capable of producing and metabolizing dopamine. Dysbiosis-induced changes in the gut microbial composition can impact dopamine production and metabolism. The influence of dysbiosis on the expression and function of dopamine receptors and transporters in the brain has been an area of growing research interest. Several studies have demonstrated that changes in the gut microbiota composition can indeed lead to alterations in dopamine receptor expression and dopamine transporter activity. One study conducted by Bercik et al. (2011) explored the effects of the gut microbiota on central levels of brain-derived neurotrophic factor (BDNF) and behavior in mice [174]. They found that germ-free mice, lacking gut microbiota, displayed altered dopamine receptor expression in specific brain regions compared with control mice with normal gut microbiota. In another study, Dinan et al. (2013) investigated the role of psychobiotics, a novel class of bacteria with potential mental health benefits, on neurotransmitter systems, including dopamine [175]. They found that certain psychobiotics, such as the Lactobacillus and Bifidobacterium strains, had the ability to modulate dopamine receptor expression and dopamine transporter activity, highlighting the impact of specific gut bacteria on the dopaminergic system [176,177,178].

### 3.3. Dysbiosis and the Immune Basis of OCD

Inflammation and immune dysregulation have been implicated in the pathogenesis of OCD. Preliminary studies have noted abnormal concentrations of IgA in children with OCD. IgA mediates microbial composition and homeostasis at the mucosal level via the prevention and promotion of bacterial growth, which both influence bacterial gene expression [115].

There is also evidence for the role of immunological processes in the pathophysiology of OCD [9,139,179,180,181]. Commonly known as the “autoimmune OCD subtype”, Pediatric Autoimmune Neuropsychiatric Disorders Associated with Streptococcal Infections, or PANDAS, is characterized by OCD that appears after a streptococcal infection, such as scarlet fever or strep throat [179]. The dramatic surge of symptoms, which happens overnight, includes motor obsessions and compulsions. In addition, children experience mood issues and anxiety attacks. In this context, a streptococcus A infection is clearly a manifestation of dysbiosis, which activates these immunological processes. Through molecular mimicry, streptococcal bacteria are believed to induce an autoimmune response that attacks the brain via neuroinflammation. Indeed, several studies have found cross-reactive antibodies targeting the brain in children with PANDAS, as well as microglial activation [9,179,182,183,184,185,186].

### 3.4. Dysbiosis and the Genetic Basis of OCD

The neurobiological basis of overactivity involving serotonin, glutamate, and dopamine is thought to be mainly rooted in genetic factors on the one hand, with a heritability of 27–65%, and environmental factors on the other [187]. Twin studies have shown that OCD can run in families [10,186]. Epigenetic alterations have been suggested to be particularly relevant in OCD. Investigations into the peripheral DNA methylation signatures of OCD are scarce, but DNA methylation patterns have been described [186].

Among the genes under investigation, notable examples include serotonergic genes (HTR2A, 5HTTLPR, SLC6A4), glutamatergic genes (SLC1A1, DLGAP3, SAPAP3), and dopaminergic genes (SLC6A3, DRD4) [10,11,187,188,189]. Recently, there has been a growing focus on the role of epigenetic mechanisms such as gene methylation, histone deacetylation (HDAC), and histone acetylation (HAT) in psychiatric disorders. Efforts have been made to develop treatment strategies targeting these mechanisms. Studies comparing DNA methylation patterns in OCD patients and control groups have revealed distinct methylation profiles in the promoter regions of genes such as MAOA, GABA, MOG, BDNF, LEPR, OXTR, SLC6A4, and SLC6A3 [190]. In a recent study, a statistically significant correlation was observed between certain obsessions/compulsions and polymorphisms in HDAC2, HDAC3, and HDAC4 in an OCD group (*p* < 0.05) [191]. Indeed, gut microbes have been found to inhibit the histone deacetylase, an enzyme that facilitates the transcription of genes by removing the acetyl group from histone proteins on DNA [192].

Other genes have been found to be associated with OCD in some studies, such as the oxytocin receptor (*OXTR*) gene [192,193,194,195,196]; the monoamine oxidase A (*MAOA*) gene [197]; the brain-derived neurotrophic factor (*BDNF*) gene [198]; the gamma-aminobutyric acid B receptor 1 (*GABBR1*), estrogen receptor 1 (*ESR1*), and myelin oligodendrocyte glycoprotein (*MOG*) genes; and, again, the brain-derived neurotrophic factor (*BDNF*) gene [199].

The growing body of evidence implicating gut microbes in epigenetics places dysbiosis at the center of our model. However, not all clinical observations can be explained by the genetic hypothesis alone. For example, several studies failed to find significant associations between the dopamine transporter gene (SLC6A3) and OCD [200,201,202,203] or dopamine receptor 2 (DRD2) and the dopamine receptor 3 (DRD3) and OCD [202,204,205]. This suggests that other mechanisms are involved in the observed increase in dopamine in the CSTC circuit.

### 3.5. Dysbiosis and the Environmental Basis of OCD

In this proposed model, childhood trauma and stressful life events can modulate the gut microbiota composition and thus trigger dysbiosis. This has been proposed to happen via the hypothalamic–pituitary axis [37,38,39,206,207]. Lifestyle changes, particularly diet, and the recurrent use of antibiotics in the early stages of life can be determinants in the early development of the gut microbiota and the development of neurodevelopmental disorders [207,208,209,210]. Indeed, gut microbiota and brain maturation, including myelination, occur synchronically between birth and three years of age, and the gut microbiota has been found to play a critical role in myelination [211,212]. The gut microbiota includes bacteria that can synthesize various neurotransmitters besides serotonin. For instance, Lactobacillus and Bifidobacterium produce GABA, Escherichia, and Bacillus; Saccharomyces spp. generate norepinephrine; Bacillus synthesizes dopamine; and Lactobacillus produces acetylcholine [213,214,215]. Thus, the disruption of the microbiota in the early years can impact the normal functioning of the gastrointestinal tract and affect the overall health of the individual, and it is likely to elevate the occurrence of diverse mental disorders [27,142,216,217].

## 4. Microbial Reprogramming Strategies

### 4.1. Prebiotics, Probiotics, and Postbiotics

If dysbiosis is a central element in the development of OCD, then it would be expected that the manipulation of the gut microbiota might influence its occurrence and offer potential options for its treatment. This does in fact appear to be the case, and in this section, we gather clinical evidence of the use of probiotics and fecal microbiota transplants in the treatment of OCD.

A probiotic is a live organism that, when ingested in adequate amounts, exerts a health benefit on the host [218]. Probiotics use dietary fibers or resistant starch as nutrient sources (or prebiotics) to produce beneficial metabolites (postbiotics). The term synbiotic is used to refer to the mixture of both prebiotics and probiotics [219]. Dietary fibers and resistant starch, therefore, play an essential role in fermentation and postbiotic production [220,221,222]. Westernized diets are characterized by a relatively low intake of dietary fiber, which could explain the presence of dysbiosis in most modern diseases and disorders. Dietary fibers also include plant-based carbohydrates, such as polyphenols, and non-carbohydrate compounds, such as lignin. Probiotics such as Lactobacillus, Bifidobacterium, and Akkermansia can use these compounds to produce SCFAs, which, in turn, promote various beneficial effects in the host [143,223,224,225,226,227,228].

Over the last decade, a number of studies have shown promising results for the use of probiotics in the treatment of OCD. However, while a growing number of studies have investigated the potential value of probiotics in treating autism and ADHD, investigations of probiotic interventions for OCD are still at their very early stages, with most studies being performed on animal models.

Kantak et al. (2014) found that two-week pretreatments with *Lactobacillus rhamnosus* GG had the ability to reduce obsessive–compulsive disorder in mice. The results were comparable to treatment with fluoxetine [228].

In 2018, Tabouy et al., using Shank3 KO mice (a model used to study neurodevelopmental disorders such as autism), found *Lactobacillus reuteri* to be in a decreased relative abundance in the Shank3 KO. The treatment of Shank3 KO mice with *Lactobacillus reuteri* induced a significant decrease in repetitive behaviors in both males and females [229].

In a study conducted by Szklany et al. (2020), male mice receiving (from the day of birth onwards) a prebiotic mixture composed of short-chain galactooligosaccharides (scGOS) and long-chain fructo-oligosaccharide (lcFOS) exhibited changes in the serotonergic system [230]. These neurological modulations were associated with behavioral changes, such as a reduction in anxiety and repetitive behavior during development and increased social interest in adulthood compared with mice fed a control diet. The brains of the treated group exhibited altered mRNA expression in astrocytic glial fibrillary acidic protein and microglial integrin alpha M. There was also enhanced mRNA expression in BDNF in the prefrontal cortex. Additionally, analysis of the cecal content of the treated animals revealed relatively increased levels of SCFA, such as butyric acid, and decreased levels of valeric, isobutyric, and isovaleric acid [230].

Another animal study conducted by Sanikhani et al. (2020) demonstrated the effectiveness of *Lactobacillus casei* Shirota in treating OCD in a rat model. After daily administrations of *L. casei* Shirota (10^9^ CFU/mL for four weeks), the probiotic showed beneficial effects, possibly effected through the modulation of genes related to serotonin. Following concurrent treatment with *L. casei* Shirota and fluoxetine, the expression level of Bdnf significantly increased, while the expression of Htr2a (serotonin receptor 2A) decreased in the orbitofrontal cortex tissues of all rats involved in the study [149].

In 2021, Sunand et al. [231] found that selected probiotic strains and complex treatments with probiotics significantly ameliorated microbial diversity; repetitive behaviors; and the concentrations of NF-a, BDNF, and 5-HT.

In 2022, Alghamdi et al. [232], using an animal model of autism induced by propionic acid, found that subjects with cognitive dysfunction had altered levels of neurotransmitters in their brains. However, in the group of animals treated with probiotics, neurotransmitter levels were 1.2-fold higher compared with the control group. In the same study, the alpha-melanocyte-stimulating hormone (α-MSH) was monitored. α-MSH acts on melanocortin type 4 receptors (MC4R), a receptor that interacts with neurochemical systems that regulate socioemotional behaviors, including oxytocin and dopamine. Oxytocin can influence social cognition by modulating various neurochemical systems, including serotonin, glutamate, dopamine, and GABA neurotransmitters in specific brain regions, such as the hypothalamus, amygdala, and hippocampus. The study observed significantly lower levels of α-MSH in animals treated with propionic acid compared with the controls. However, this effect was reversed by the administration of bee pollen and a mixed probiotic bacteria preparation called ProtexinR, which contains beneficial bacteria such as *Bifidobacterium infantis*, *Bifidobacterium breve*, *Lactobacillus acidophilus*, *Lactobacillus bulgaricus*, *Lactobacillus casei*, *Lactobacillus rhamnosus*, and *Streptococcus thermophiles*. The concentration of the mixed probiotic bacteria in ProtexinR was 1 billion CFU per gram [232].

Pochakom (2022) investigated supplementation with *Lacticaseibacillus rhamnosus* HA-11 (Lr) and *Ligilactobacillus salivarius* HA-118 (Ls) in the BTBR T+ *Itpr3tf*/J (BTBR) mouse model of autism (10^9^ CFU/mL in drinking water for 4 weeks) [233]. Supplementation with Lr, but not Ls, increased the microbial richness and diversity and increased the concentrations of beneficial neuroactive compounds, such as 5-aminovaleric acid and choline. Both the Lr and Ls treatments reduced behavioral deficits in social novelty preference, but no changes in hyperactivity or repetitive behaviors were observed [233]. This suggests that not all probiotic microbes result in the same outcomes, and a more complex mix of microbes might actually be required to target various behaviors.

Sen et al. 2022 [234] found that the daily oral administration of *Blautia stercoris* MRx0006 attenuated social and repetitive behaviors in a mouse model of autism. The study showed that MRx0006 increases the expression of oxytocin and its receptor in hypothalamic cells in vitro and hypothalamic oxytocin mRNA in mice while altering the metabolome profile. It was proposed that biotherapy using *Blautia stercoris* would be a viable treatment option for autism.

Studies on human subjects are rarer but encouraging. A double-blind randomized controlled trial focused on the effect of a synbiotic called Synbiotic 2000, composed of three anti-inflammatory lactic acid bacteria and four anti-inflammatory fibers, on patients with ADHD [218]. One of the measured outcomes was repetitive behavior. Synbiotic 2000 reduced both the total score of autism symptoms and restricted, repetitive, and stereotyped behaviors, as compared with a placebo [229]. Similarly, in a case report on a child with autism, *Sacharomyces boulardii* was shown to reduce OCD behavior [235].

### 4.2. Fecal Microbiota Transplants

Fecal microbiota transplantation (FMT), or the transfer of fecal matter from a healthy donor to a patient, has emerged as another promising therapeutic approach for restoring a healthy gut microbiome and achieving beneficial effects in various diseases [236]. Currently, there are no FMT studies that have been performed specifically to treat OCD. However, several studies have noted significant changes in microbial ecology, metabolism, and behavior observed in patients after FMT, most of them providing strong support for FMT as a therapeutic method to treat OCD [237,238,239,240,241,242].

Kang et al. (2017) published an important follow-up after the publication of the first clinical trial results using FMT on autistic children [243]. Spectacular improvements were observed in GI symptoms, autism-related symptoms, and gut microbiota diversity with a higher abundance of Bifidobacteria and Prevotella, and these were sustained after two years [237]. The autism-related symptoms even exhibited further improvement, suggesting that the fecal transplants might have initiated further changes during the two-year period. These findings underscore the long-term safety and effectiveness of FMT as a potential therapy for gut-dysbiosis-associated disorders. Although the focus of the study was not OCD behavior, it is particularly relevant considering the close relationship between gut dysbiosis and brain dysfunction. For example, Kilinçarslan et al. (2020) found that the severity of several factors, including obsession, decreased after FMT in patients with inflammatory bowel disease [244]. This suggests that the restoration of a healthy gut microbial community through FMT can have positive effects on psychological symptoms associated with certain diseases. Alghamdi et al. (2022) conducted a study in a rodent model of autism and included the use of FMT from healthy donor rats, which resulted in a significant increase in α-MSH levels by 2.7 fold (compared with 1.2 fold for probiotics) and an increase in the brain levels of neurotransmitters (1.6 fold) and substance P (2.2 fold) to above that of the controls [232]. These results suggest that FMT might be superior to probiotics in initiating metabolic changes; however, further clinical studies are needed to compare both the efficacy and safety of FMT and probiotics.

Although not focusing on OCD but on autism, a very interesting recent study by Wang et al. (2023) highlights important changes after fecal transplants [245]. Fecal microbiota samples from ASD children and healthy donors were transplanted into a mouse model of ASD. The researchers conducted 16S rRNA gene sequencing on fecal samples and untargeted metabolomic analysis on samples to identify differences in gut microbial communities and metabolic pathways related to ASD behaviors. mRNA sequencing analysis was also performed on colon and brain tissues after sacrificing the animals to identify enriched signaling pathways and potential molecular mechanisms. The study revealed metabolite changes related to serotonergic and glutamatergic synapse pathways. They also demonstrated that these were associated with behavioral changes in ASD: there was an increase in ASD-like behaviors in mice that received FMT from ASD donors but a decrease in such behaviors in mice that received FMT from healthy donors. Indeed, the colonization of certain bacterial genera, such as Bacteroides, Odoribacter, Turicibacter, and Alistipes, was correlated with an improvement in behavior after FMT, but this did not specifically point to OCD behavior. However, the changes in serotoninergic and glutamatergic pathways might also predict positive outcomes for future OCD studies.

The close link between gut dysbiosis and brain function underscores the importance of targeting the gut microbiota for therapeutic interventions. FMT offers a unique opportunity to restore a healthy gut microbial community and potentially alleviate symptoms associated with various disorders. These findings highlight the potential benefits of FMT in improving mental health conditions and support the further exploration of this therapeutic approach.

## 5. Discussion

The correlation between gut dysbiosis and the vast majority of modern diseases is now firmly established [134,141,246,247,248,249,250,251,252]. The gut microbiome might actually play a significant role in the development and manifestation of OCD, providing a comprehensive explanation for the multiple factors previously associated with the disorder. The gut microbiome can influence genetic, neurobiological, and environmental factors indirectly, thereby impacting the pathophysiology of OCD.

We have seen that SCFAs can improve gut–brain health via a number of pathways, including maintaining the gut barrier integrity; producing mucus; protecting against inflammation; and communicating with the brain via the vagus nerve and neurohormones [34,253,254]. However, the gut’s bacterial composition is determined by multiple factors, including genetics, immune status, drugs (e.g., metformin), antibiotic courses, diet, pollutants, etc. [207]. Thus, studies investigating the gut microbiota composition must consider multiple variables. Furthermore, the various methods for the collection, storage, and handling of microbiological materials add even more variability to these studies [255,256]. Microbial reprogramming strategies, using either probiotics or FMT, may also encounter significant challenges arising from interindividual variations and even temporal variations within a single individual.

The host’s genetic background can modulate bacterial colonization, particularly genetic variants such as single nucleotide polymorphisms, which could explain the interpersonal variability in circulating levels of SCFA observed after fiber intake. Variations in genes coding the receptors of SCFAs, such as GPR41, GPR43, and GPR109A, have been proposed to have a significant impact on metabolism in general [96,257,258]. Additionally, genes responsible for transportation, such as the SLC16A family of monocarbohydrate transporters, effector genes like MUC2 involved in colon mucus production, and regulatory genes like NRF2, which regulate the expression of proteins related to antioxidant defense mechanisms, may have significant implications for health outcomes. These effects could arise from the compromised absorption of short-chain fatty acids or their intracellular functions [259].

The impact of probiotics on human health has been studied through clinical trials and resulted in numerous suggested health indications and claims [260,261,262,263,264,265]. Nevertheless, there are also studies with contradictory findings, resulting in conflicting, ambiguous conclusions regarding the efficacy of probiotics [266,267]. One of the main reasons for these conflicting results (and also the main challenge for future studies) is that, in contrast to animal models, humans exhibit significant heterogeneity in terms of diet, age range, genetic background, and gut microbiome composition [268,269]. As a result, they may respond differently to the same probiotic intervention. In fact, several studies on probiotics have emphasized the importance of precision in considering host-related factors, microbiome characteristics, and dietary influences, as these factors play a crucial role in determining the varied outcomes observed [270,271]. More specifically, the extent of gut colonization by probiotics can vary significantly between individuals. This variability in colonization levels can contribute to the diverse effects of probiotics on both the hosts themselves and their gut microbiomes [272]. This is understandable since pre-existing microbes can influence each other’s growth and that of the newly ingested microbes. If dysbiosis is already present, such as in atopic dermatitis or milk hypersensitivity, dysbiosis can alter the effects of the probiotic intervention on the host [273,274]. These permissive microbiomes are also more susceptible to compositional and functional changes in response to probiotics, resulting in the distinct enrichment of pathways in the gut [275]. Microbiomes that facilitate the colonization of probiotic bacteria are associated with improved clinical responses in various models of colitis and depression [276,277,278].

Therefore, differences in the initial conditions of the host and their gut microbiome, as well as variations in environmental exposures, can lead to contrasting outcomes between individuals who receive the same probiotic supplement [279,280]. In addition, for in vitro studies, the characteristics of probiotic bacteria, such as adhesion, hydrophobicity, and autoaggregation, may vary depending on the source from which they were isolated [281,282].

Environmental factors, as described in our model, influence the gut microbiome and, thus, the response to probiotics. Dietary polyunsaturated fatty acids, for instance, have been found to modulate the adhesion of probiotics in laboratory settings [279]. Similarly, diet can impact clinical outcomes, as preterm infants fed with human milk demonstrate a reduced risk of late-onset sepsis and a shorter time in achieving full enteral feeding compared with formula-fed infants [283].

Consequently, we consider FMT interventions to be an option with less variability in terms of results. This is because, with FMT, an entire community of microbes, including fungi, is transplanted. However, the downside of this is that we do not currently have enough data to predict, manage, or control the eventual risks patients are exposed to when transplanted with the gut microbiome of another healthy individual. Indeed, FMT has shown promise with positive outcomes for various diseases [237,240,254,284,285]. However, the escalating problem of antibiotic resistance poses a threat to the use of FMT. Sample screening must follow rigorous guidelines as antibiotic resistance becomes a criterion for donor stool selection. For FMT to become a successful approach in disease treatment and management, advances are necessary in defining the composition of fecal samples and methods of administration. There must also be a shift toward personalized fecal sample selection. The future of a safe FMT probably resides in our ability to further elucidate what the phrase “healthy microbiome” really means. New analytical techniques, such as machine learning, might become necessary tools to integrate into omics studies in order to find the best FMT for OCD [286].

## 6. Conclusions

We presented a model where dysbiosis plays a pivotal role in the pathogenesis of OCD. To validate this model, and to shed more light on the potential role of gut microbes in the pathogenesis and treatment of OCD, more clinical studies are needed. Exploring the gut microbiome as a target for intervention in OCD holds promise for several reasons. Indeed, this is an opportunity to address the limitations of current treatments and potentially improve treatment outcomes for individuals who experience resistance to current approaches. In addition, the gut microbiome represents a modifiable factor that can be influenced in various ways, including dietary interventions, probiotics/prebiotics, and even fecal microbiota transplantation.

Although the use of probiotics and FMT in medicine has been used empirically for centuries, it was only once research acknowledged the importance of the role of the gut microbiota in health and diseases that clinical studies started developing its use. Most studies have used Bifidobacteria, such as B. longum, B. breve, and B. infantis, and Lactobacilli (L. helveticus and L. rhamnosus), with doses between 10⁸ and 10¹⁰ colony-forming units for about 4 weeks. These probiotics have shown efficacy in improving psychiatric disorder-related behaviors including anxiety, depression, ASD, ADHD, and OCD.

However, research in this area is still in its early stages, and more studies are needed to optimize the methods and assess the efficacy and safety of microbial reprogramming in OCD. Further investigation and clinical trials will pave the way for more personalized and effective interventions for individuals with OCD, perhaps through the development of disorder-specific probiotic mixtures, making microbiome therapeutics a significant part of the precision medicine field.

Given the significant impact of OCD on individuals’ lives and the limited effectiveness of current treatments, it is crucial that we urgently direct our efforts toward studying the role of the gut microbiota in OCD. By bridging the knowledge gap and conducting rigorous clinical interventions, we can uncover new insights into the pathogenesis of OCD and develop innovative therapeutic strategies. These advancements have the potential to revolutionize the field, offering hope and improved outcomes for individuals affected by this debilitating disorder. We call upon the scientific community to prioritize and support research in this vital area, as it represents a crucial step toward personalized and effective interventions in OCD.

## Figures and Tables

**Table 1:** Dysbiosis in OCD.

	Sample Type	Observations	Year	Ref.
36 OCD patients and 35 controls	Blood	ELISA: - Significantly higher mean serum claudin-5 in OCD may contribute to the role of the blood–brain barrier in the pathogenesis of OCD. - Serum zonulin level was not different from the control group in OCD patients.	May 2022	[146]
64 OCD patients (30 women and 34 men) and 51 controls (31 women and 20 men)	Saliva Blood	Human study rRNA-gene-based PCR sequencing: - Significant increase in *Actinobacter* and *Firmicutes* in OCD. - Significant decrease in *Fusobacteria*-to-*Actinobacteria* ratio in OCD patients compared with controls. - No differences in *Bacteroidetes*, *Proteobacteria,* or *Fusobacteria.* - No differences were observed in *Firmicutes*-to-*Bacteroidetes* ratio in OCD and controls. Quantitative methylation analysis, PyroMark Q24: - Significant correlation between the levels of DNA methylation and the abundance of *Actinobacteria* but not between the epigenetic mark and *Firmicutes* levels.	Mar 2022	[147]
6 Wistar rats (ISO with anxious-like phenotype and controls)		Animal study LC–MS/MS analysis: - Total SCFA levels significantly reduced one week after isolation in ISO rat feces when compared with the control group. - Significant decrease in butyrate concentration, whereas acetate, propionate, and valerate levels were not affected by social isolation.		
38 OCD patients (20 females, 18 males) and 33 controls (18 females, 15 males)	Oropharyngeal swab samples (tonsils, throat) Fecal	LEfSe analysis: - Significant increase in the relative abundance of *Rikenellaceae (Alistipes* genus) in OCD. - Significant decrease in the levels of *Prevotellaceae* in OCD samples compared with controls. - Different distribution of order Clostridiales: increase in *Oscillibacter, Anaerostipes,* and *Flavonifractor and decrease in Agathobacter, Coprococcus, Lachnospira, Howardella, Romboutsia, Butyricicoccus,* and *Clostridium* compared with controls. - Depletion of *Lachnospira pectinoschiza* correlated with OCD severity on the obsession subscale. - No significant difference before vs. after behavioral therapy.	Jan 2022	[135]
28 Wistar rats: compulsive high drinkers (HD) or low drinkers (LD)	Fecal Blood	PCR–DGGE analysis: - Lower bacterial diversity in compulsive HD rats as compared with LD rats, irrespective of diet. - TRP depletion induced a reduction in bacterial evenness in HD rats. - TRP depletion induced a reduction in peripheral plasma 5-HT levels in both HD and LD rats. - Possible implication of reduced microbial diversity in compulsive behavior via the serotonergic system.	May 2021	[148]
4 groups of 6 male Wistar rats, OCD-induced with D2 agonist quinpirole, and 1 group of 6 rats as control	Brain tissue	Intervention: Treatment with L. casei Shirota (10^9^ CF/g, daily for 4 weeks) and fluoxetine. Behavioral test: - Decrease in OCD-like behaviors in OCD rats after being fed with *L. casei* Shirota (explored all boxes, including center), similar to negative control and fluoxetine-treated. qPCR: - Increase in *Bdnf* expression in brain tissue in OCD rats after being fed with *L. casei* Shirota, similar to negative control and fluoxetine-treated. - Decrease in *Htr2a* expression in OCD rats after being fed with *L. casei* Shirota, similar to negative control and fluoxetine-treated.	Dec 2020	[149]
21 OCD 22 controls	Blood Fecal	- Lower species richness/evenness (α-diversity, Inverse Simpson) and lower relative abundance of three butyrate-producing genera (Oscillospira, Odoribacter, and Anaerostipes) in OCD. - Increased mean CRP in OCD, with moderate-to-strong associations with symptomatology. - No change in IL-6 or TNF-α.	Oct 2020	[138]
11 deer mice (8 females, 3 males) per group 2 groups: large nest-building natural OCD (LNB) and normal nest building (NNB)	Fecal	16sRNA: - Normal-phenotype animals showed a higher loading of Prevotella and Anaeroplasma. - Natural OCD phenotype demonstrated a higher loading of Desulfovermiculus, Aestuariispira, Peptococcus, and Holdemanella.	Mar 2020	[150]
21 OCD patients and 22 controls	-	- Increased gastrointestinal symptom severity in OCD compared with controls. - Increased prevalence of IBS in OCD compared with controls.	Nov 2019	[137]
30 PANS/PANDAS patients	Fecal	16sRNA: - Significant increase in Bacteroidetes in PANS/PANDAS (*Bacteroides*, *Odoribacter*, and *Oscillospira* proposed as biomarkers) - Negative correlation between genera belonging to Firmicutes phylum and anti-streptolysin O - Targeted metagenomics: - Increase in several pathways concerning the modulation of the antibody response to inflammation. - Decrease in pathways involved in brain function.	Apr 2018	[135]

## Data Availability

No new data were created or analyzed in this study. Data sharing is not applicable to this article.
